# Gout and Hyperuricemia: A Narrative Review of Their Comorbidities and Clinical Implications

**DOI:** 10.3390/jcm13247616

**Published:** 2024-12-13

**Authors:** Janis Timsans, Antti Palomäki, Markku Kauppi

**Affiliations:** 1 Department of Rheumatology, Päijät-Häme Central Hospital, Wellbeing Services County of Päijät-Häme, 15850 Lahti, Finland; markku.kauppi@paijatha.fi; 2 Faculty of Medicine and Health Technology, Tampere University, 33100 Tampere, Finland; 3 Centre for Rheumatology and Clinical Immunology, Turku University Hospital, 20521 Turku, Finland; 4 Department of Medicine, University of Turku, 20014 Turku, Finland; 5 Clinicum, Faculty of Medicine, University of Helsinki, 00014 Helsinki, Finland

**Keywords:** gout, hyperuricemia, renal hyperuricemia, metabolic hyperuricemia, gouty arthritis, comorbidities

## Abstract

Gout is the most common form of inflammatory arthritis, caused by the deposition of monosodium urate crystals in the joints due to elevated serum uric acid levels. Its prevalence and associated healthcare burden have been rising in recent decades, a trend expected to continue. It is crucial to recognize that gout and hyperuricemia are not merely causes of painful joint flares, but systemic metabolic disorders linked to a broad spectrum of comorbidities such as cardiovascular diseases, chronic kidney disease, diabetes, insulin resistance, steatotic liver disease, osteoarthritis, and respiratory and eye diseases. Numerous risk factors for gout and hyperuricemia have been identified, with recent research uncovering further associations with other conditions. To optimize patient outcomes, gout and hyperuricemia must be addressed through a holistic approach that accounts for these risk factors while providing comprehensive management of related comorbidities affecting various organ systems. This review summarizes the current knowledge on the risk factors, comorbidities, and clinical implications of gout and hyperuricemia. Future research should focus on improving patient outcomes by tailoring treatments individually and addressing the underlying metabolic comorbidities of gout with multimodal treatment.

## 1. Introduction

Gout, the most common inflammatory joint disease worldwide [1], is characterized by the deposition of monosodium urate (MSU) crystals in joints and surrounding tissues, causing acute pain and inflammation. Recognized since ancient times [2] as the “disease of kings” due to its associations with lifestyle factors, gout’s pathogenesis centers on elevated serum uric acid (SUA) levels, or hyperuricemia, which is now recognized as a primary etiological factor for crystal deposition. This link was first suggested by Antoni van Leeuwenhoek nearly 350 years ago when he identified uric acid crystals in a gouty tophus [3]. Despite modern diagnostic and therapeutic advances, gout remains underdiagnosed, misdiagnosed, and suboptimally treated worldwide [4], contributing to its rising incidence and increased healthcare burden [5].

The importance of managing gout and hyperuricemia extends beyond simply reducing painful joint flares. Hippocrates, who described gout around 400 BC, observed potential associations with broader health issues [6], a notion that research in the 20th and 21st centuries has supported and expanded. Today, gout and hyperuricemia are recognized as systemic metabolic disorders associated with a range of comorbidities, including cardiovascular diseases, chronic kidney disease, metabolic syndrome, and hepatic steatosis. These associated conditions, if left unaddressed, can significantly impact the patient quality of life and long-term health outcomes. Thus, the effective management of gout necessitates a comprehensive approach that considers the underlying metabolic disturbances and comorbid conditions, rather than focusing solely on joint pain management.

This review aimed to summarize the current knowledge on the risk factors and pathophysiological drivers of hyperuricemia and gout, examine the comorbidities linked to these conditions, and discuss the clinical implications for optimizing patient care. In doing so, we highlighted the need for a holistic approach that addresses both gout itself and its broader health impacts. To the best of our knowledge, this is the first comprehensive review on gout and hyperuricemia comorbidities that acknowledges the novel distinction between the etiological types of hyperuricemia (renal versus metabolic) and examines the known differences between these two types.

## 2. Uric Acid and Formation of Monosodium Urate Crystals

Uric acid is the end product of both exogenous purine intake and endogenous purine metabolism [7]. An elevation in the concentration of serum uric acid (SUA) above a certain threshold is a necessary condition for the formation of MSU crystals. The in vitro solubility limit of MSU is approximately 404 µmol/L (approximately 6.8 mg/dL) [8]. There are, however, many factors that have an impact on this threshold. An acidic environment seems to promote the crystallization of MSU [9]. Temperature affects MSU crystal formation—in vitro studies conducted in aqueous solutions have indicated that a temperature decrease of just 2 °C (from 37 °C to 35 °C) is enough to reduce the solubility threshold of urate from 404 to 360 µmol/L (approximately 6 mg/dL) [10]. In vivo, factors related to synovial fluid and cartilage likely modulate MSU crystallization [11].

The aforementioned factors complicate the definition of hyperuricemia. The same level of SUA may be critical for MSU crystal formation in some situations, but not in others. Conditions associated with acidosis—such as respiratory insufficiency, renal failure, strenuous exercise, and alcohol consumption—are likely to lower the crystallization threshold of urate. The link between these conditions and gout attacks has already been identified. Peripheral sites with lower temperatures are more prone to MSU crystal formation than warmer areas [12]. The temperature of the first metatarsophalangeal joint, the most common site for gout attacks, is approximately 32 °C [13].

There is indeed no international consensus regarding the SUA cut-off in the definition of hyperuricemia. In many sources, hyperuricemia is defined as an SUA level >360 μmol/L (approximately 6 mg/dL) [14,15,16], whereas others use an SUA level of 420 μmol/L (approximately 7 mg/dL) as a cut-off [17,18]. Numerous sources define hyperuricemia as an SUA level >360 μmol/L (approximately 6 mg/dL) in women and >420 μmol/L (approximately 7 mg/dL) in men [19,20,21], even though there is no reliable evidence that the precipitation threshold of MSU crystals would differ in men and women.

The variability in hyperuricemia thresholds arises from differing approaches to defining SUA levels. Some experts support using thresholds based on population distributions, where a significant proportion of individuals have SUA levels above the uric acid crystallization point, leading to higher SUA thresholds. This population-based approach often results in different cut-offs for men and women, reflecting the generally higher SUA levels in men. Alternatively, a clinically oriented definition of hyperuricemia focuses on the uric acid crystallization threshold, approximately 360 µmol/L (about 6 mg/dL) in peripheral body areas, which more directly aligns with the risk of gout and crystal formation.

Hyperuricemia does not necessarily lead to gout. It has been reported that only up to 36% of hyperuricemic individuals develop gout attacks [22]. Hyperuricemia is positively associated with incident gout in a dose-dependent manner [23,24]. It has, however, been found that only about half of the individuals with SUA concentrations of ≥600 μmol/L (approximately 10 mg/dL) developed clinically evident gout over a 15-year period [23]. It is not completely clear why some hyperuricemic individuals develop gout attacks and others do not. The mechanisms implicated include the overstimulation of cell proliferation and inflammation, the production of genetic variance in chemotactic cytokines, and the internalization of pro-apoptotic and inflammatory factors induced by extracellular uric acid [25].

Although asymptomatic hyperuricemia represents the initial stage in the progression of gout, and both conditions share common risk factors and comorbidities, the degree to which specific risk factors and comorbidities are associated with asymptomatic hyperuricemia often differs from their association with gout. In this review, we have clarified whether the discussed factor pertains to hyperuricemia or gout to distinguish between the two conditions effectively.

## 3. Risk Factors of Hyperuricemia and Gout

A summary of the risk factors for gout and/or hyperuricemia is provided in Table 1.

### 3.1. Sex

It has long been established that gout is more prevalent in men than in women. A recent study reported that, globally, the prevalence of gout in 2020 was 3.26 times higher in males than in females [26]. This discrepancy is more pronounced in younger individuals: among those under 65 years of age, men have a four-fold higher prevalence of gout compared to women; this male-to-female ratio is 3:1 in individuals over 65 years [27]. This is due to a later onset of gout in women, which stems from the effect of estrogen in premenopausal women—estrogen enhances renal tubular excretion, effectively lowering the levels of SUA [28]. It has also been demonstrated that estradiol regulates intestinal ATP-binding cassette subfamily G member 2 (ABCG2) via the PI3K/Akt pathway, promoting urate excretion [29].

### 3.2. Age

An advancing age is closely linked to a higher risk of hyperuricemia and gout. It has been demonstrated in numerous cohorts that SUA rises with age [26,30,31]. In elderly cohorts, hyperuricemia has been found to be highly prevalent. The PolSenior study from Poland observed hyperuricemia [defined as an SUA level above 6 mg/dL (approximately 360 μmol/L) in women and 6.8 mg/dL (approximately 404 μmol/L) in men] in 28.2% of women and 24.7% of men aged 65 and older. In individuals aged 90 and above, the prevalence increased to 33.7% in women and 30.5% in men [32]. The GOAL study from Finland, which investigated individuals aged 52 to 76, found an even higher prevalence of hyperuricemia [defined as an SUA level ≥360 μmol/L (approximately 6 mg/dL)], at 48%, with 31% in women and 60% in men [33]. The prevalence of gout among hospitalized, multimorbid elderly patients in an Italian study was found to be 10% [34].

### 3.3. Body Composition

There is a strong connection between obesity and both hyperuricemia and gout. It has been demonstrated in multiple studies [23,35,36,37,38]. It has been shown that, for every 5 kg/m² increase in body mass index (BMI), the risk of developing gout rises by 55% [39]. Several studies have examined the link between abdominal adiposity, measured by the waist circumference or waist-to-hip ratio, and the risk of gout, consistently reporting an increased risk [36,40,41]. Weight loss has a protective effect against gout [36,42]. Bariatric surgery has been demonstrated to decrease the SUA levels and reduce the incidence of gout flares in the long term, even though the SUA level as well as the risk for gout flares rises in the first post-operative month [43].

Overweight and obesity raise uric acid levels through several mechanisms. Excess body fat increases the breakdown of purines, compounds found in certain foods and cells. This leads to an increased production of uric acid, as purines are metabolized into urate. Adipose tissue generates uric acid via the enzyme xanthine oxidoreductase (XOR), and this production is increased in individuals with obesity [44]. Additionally, overweight individuals often have reduced kidney function [45], leading to an impaired ability of the kidneys to excrete uric acid. Obesity is associated with low-grade chronic inflammation [46], which can contribute to metabolic changes that promote hyperuricemia. Overweight individuals also likely consume diets higher in purine-rich foods, such as red meat and sugary beverages (especially fructose), both of which are linked to higher uric acid levels [47].

### 3.4. Genetic Factors and Ethnicity

The likelihood of developing hyperuricemia and gout differs among populations based on race and ethnicity. It has been demonstrated that Black individuals have a 1.5- to 2-fold increased risk of gout compared to White individuals [48,49]. A recent study of the general United States population found that gout was 1.8 times more prevalent in Black women compared to White women and 1.3 times more prevalent in Black men compared to White men [50]. However, these associations weakened after adjusting for factors such as poverty, diet, BMI, and chronic kidney disease (CKD) in women, and for diet and CKD in men. Once all the risk factors were accounted for, the differences between racial groups were no longer significant for either sex. Similar results were observed for hyperuricemia. These findings suggest that racial disparities in gout may be largely explained by diet, social determinants of health, and CKD.

Historically, gout has been relatively rare in many parts of Asia compared to Western countries, but its prevalence has been rising in recent decades [26]. This might be due to the increasing adoption of Westernized diets and lifestyles in many Asian countries [51,52]. In the diverse population of the United States, it was demonstrated almost two decades ago that Asians were 2.7 times more likely than Caucasians to have an ambulatory care visit for gout [53]. A recent study revealed that, in 2017 to 2018, the prevalence of gout among Asian individuals in the United States exceeded that of all other racial and ethnic groups. The disparity between Asian and White individuals was not linked to socioclinical factors [54].

Oceania, particularly among Pacific Islander populations, has one of the highest prevalence rates of gout in the world [26]. Countries like New Zealand, Samoa, and French Polynesia report significantly higher rates of gout compared to other regions [55,56,57]. This elevated prevalence is thought to be influenced by a combination of genetic predisposition and lifestyle factors, such as diets high in purine-rich foods and increasing rates of obesity and metabolic syndrome.

In New Zealand, for example, gout is especially common among the Māori and Pacific Islander populations [58], where genetic factors affecting uric acid metabolism may play a significant role. These populations are particularly vulnerable to hyperuricemia and gout-related health complications.

Over 20 susceptibility genes for hyperuricemia and gout have been identified [59]. Some are linked to increased uric acid production, while others are related to enhanced reabsorption in the proximal renal tubule, reduced excretion, or other mechanisms that contribute to elevated uric acid levels and gout. Among the most studied gene families are the SLC22A, ABC, and SLC2A families, which are recognized for their role in uric acid metabolism [59].

### 3.5. Dietary Factors

Historically, gout has been closely linked to the consumption of purine-rich foods and excessive alcohol intake. Nearly two-thirds of the purines in the body are produced endogenously, while the rest, known as exogenous purines, come from food sources [60]. Purine-rich foods that have been shown to raise SUA levels include seafood, legumes, red meat, and poultry [61]. Additionally, the consumption of sugar-sweetened beverages and a high-fructose diet are associated with elevated SUA levels [62,63,64]. Alcohol consumption leads to significant increases in SUA levels [65,66,67,68]. The ethanol in alcoholic beverages significantly impacts serum urate levels by both increasing uric acid production and reducing its elimination through the urine, primarily by altering the kidney tubule function [69]. The type of alcoholic beverage seems to play a significant role in hyperuricemia risk—beer and liquor have consistently been found to markedly raise SUA levels [67,69]. However, the findings for moderate wine consumption are more mixed [70,71]. Some studies suggest that a moderate wine intake may protect against gout attacks due to wine’s antioxidant and phytoestrogen content, though the results remain somewhat conflicting [72].

A plant-based dietary pattern has been shown to be negatively associated with SUA levels [73]. A recent Mendelian randomization study found that cheese, tea, coffee, and dried fruit intake was associated with lower SUA levels, suggesting a potential protective effect against the risk of gout attacks [74]. A meta-analysis examining the effects of coffee consumption on the SUA showed that coffee significantly reduces the SUA levels; however, there were gender differences in the amount of coffee needed to achieve this effect. Women required 4–6 cups per day, while men needed only 1–3 cups per day to lower their SUA [75]. Another meta-analysis found no significant difference in the serum uric acid (SUA) levels between the highest and lowest coffee intake categories; however, it did reveal a significant inverse association between coffee consumption and the incidence of gout [76]. This finding aligns with a recently published Mendelian randomization analysis, which revealed that coffee consumption can causally reduce the risk of gout and may do so independently of SUA levels [77]. A prospective cohort study of 447,658 UK Biobank participants initially free of gout revealed a strong nonlinear association between tea or coffee consumption and reduced gout risk, with significant reductions observed at approximately six cups of tea or three cups of coffee per day [78]. A recent study conducted in the American population with chronic kidney disease found an inverted U-shaped relationship between coffee consumption and SUA levels [79].

### 3.6. Medication

Many pharmacologic agents influence SUA levels. The drugs that increase SUA levels include diuretics (particularly thiazide diuretics), low-dose aspirin, nicotinic acid, testosterone, xylitol, the anti-tubercular drugs pyrazinamide and ethambutol, and some immunosuppressants, such as ciclosporin, tacrolimus, and mizoribine [80]. Cytotoxic chemotherapy may induce tumor lysis syndrome, which leads to an increase in SUA levels due to the massive breakdown of tumor cells [81]. Tumor lysis syndrome has also been reported following treatment with dexamethasone, zoledronic acid, thalidomide, bortezomib, rituximab, and ibrutinib [82].

Several drugs prescribed for indications other than treating hyperuricemia decrease the SUA levels. These include losartan, calcium channel blockers, high-dose aspirin, leflunomide, statins, fenofibrates, sodium glucose co-transport 2 (SGLT2) inhibitors, and estrogen [83].

## 4. Comorbidities Associated with Gout and Hyperuricemia

**Figure 1:**
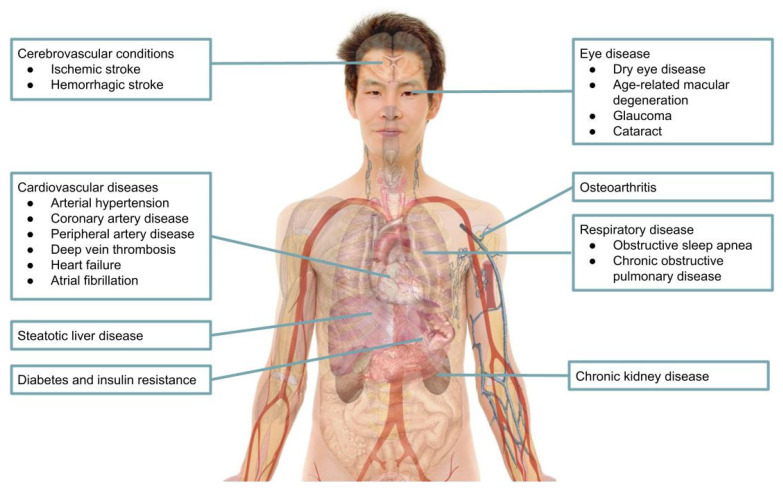
Comorbidities associated with gout and hyperuricemia. Source of image: Wikimedia Commons. Labels added by the authors of this article.

Figure 1 summarizes the comorbidities associated with gout and hyperuricemia.

Many observational studies have identified numerous conditions associated with hyperuricemia and/or gout. However, comprehensive data on causality remain lacking. Some conditions may cause hyperuricemia, while others may result from it. In some instances, there could be a bidirectional relationship, or the conditions may coexist without any causal link.

Elevated SUA levels have been recognized as a risk factor for all-cause mortality, as well as for cardiovascular, renal, and respiratory-related deaths in many observational studies [33,84,85,86,87,88,89,90,91,92,93,94,95,96]. Several studies suggest that the relationship between SUA levels and mortality follows a U-shaped pattern, indicating that both extremely high and extremely low SUA levels may be detrimental [88,89,90,91,92,93].

Recent research has yielded interesting insights into the role of hyperuricemia etiology in mortality. The Finnish GOAL study demonstrated that the risk of mortality, and especially cardiovascular mortality, related to hyperuricemia is higher in individuals with normal renal function compared to those with impaired renal function. This suggests that hyperuricemia caused by reduced glomerular filtration (renal hyperuricemia) may be less harmful than hyperuricemia resulting from excessive uric acid production (metabolic hyperuricemia) [97,98]. A similar finding was reported in the Italian URRAH study, which showed that a higher SUA-to-creatinine ratio was independently correlated with an increased mortality risk. This suggests that hyperuricemic individuals with lower serum creatinine levels (indicative of better renal function) face a higher mortality risk compared to those with higher serum creatinine levels (indicative of reduced renal function) [99].

Recently, there have been meta-analyses published on the impact of gout on all-cause and cause-specific mortalities. The all-cause mortality was 23% higher in individuals with gout compared to those without gout [100]. An increase in mortality from any cardiovascular disease (CVD) in individuals was found to be 30% higher in persons with gout compared to those without gout. The increase in mortality was 28% for coronary heart disease and 13% for myocardial infarction [101]. Gout also raised the infection mortality by 24% and the digestive system disease mortality by 42% [100].

### 4.1. Cardiovascular Diseases

#### 4.1.1. Arterial Hypertension

There is an association between hypertension and hyperuricemia [102] as well as between hypertension and gout [1]. An analysis of the 2007–2008 NHANES survey in the United States revealed that 74% of the 3.9% of individuals with gout also had hypertension. The likelihood of having hypertension was 4.2 times higher compared to age- and sex-matched controls [103]. A bidirectional Mendelian randomization study using data from the Taiwan Biobank found that the liability of gout has a causal effect on the development of hypertension, whereas the liability of hypertension does not have a causal effect on gout [104]. In the Rotterdam study, it was found that a higher uric acid genetic risk score is associated with lower systolic and diastolic blood pressure [105]. It is important to note, however, that Mendelian studies focus on gene-related associations. While hyperuricemia has a significant genetic component, it is largely influenced by lifestyle factors and diet. Further research is needed for a definitive determination of the causal role of hyperuricemia and gout on the development of hypertension.

#### 4.1.2. Arterial Diseases

Numerous studies indicate that hyperuricemia negatively affects the onset, progression, and prognosis of coronary artery disease [106]. A meta-analysis found that hyperuricemia is associated with an increased risk of coronary heart disease morbidity, with an adjusted risk ratio of 1.13 [107]. Another meta-analysis examining the incidence and prevalence of CVD in individuals with gout found a pooled prevalence of myocardial infarction at 2.8% among gout patients [108]. A Mendelian randomization study investigating the causal relationships between hyperuricemia, gout, and CVD found a significant positive association between a genetic predisposition to hyperuricemia and CVD in both one-sample and two-sample analyses. However, a genetic liability for gout was not associated with CVD [109].

Cardiovascular events often occur shortly after gout flares [110,111,112], suggesting that severe inflammation may contribute to these events and that preventing flares could play a key prognostic role [113]. This hypothesis is supported by studies showing that a negative cardiovascular outcome reduction with urate-lowering therapy (ULT) occurs after prolonged use, rather than during the initial stages of treatment [114]. This suggests that the cardiovascular prognosis improves once gout flares are effectively controlled.

A recent review by Leung et al. focused on peripheral arterial disease (PAD) in patients with hyperuricemia and gout [115]. Their research indicated that individuals with hyperuricemia or gout are at an increased risk of developing PAD. The association between elevated SUA levels and PAD is stronger than that between gout and PAD. However, it remains unclear whether an elevated SUA level is a contributing factor or simply a marker for PAD.

An elevated SUA level and gout have been demonstrated to be linked to the development of aortic aneurysms [116,117]. An increased level of SUA serves as an independent predictor of hospital mortality in patients with a type A acute aortic dissection [118]. There have been aortic MSU deposits found on dual-energy computed tomography (DECT) images in multiple studies [119,120,121,122]. In a recently published Mendelian randomization study, serum urate was identified as a risk factor for an aortic aneurysm, and this causal relationship was found to be mediated through high-density lipoprotein cholesterol, which accounted for 10.2% of the effect; there was, however, no causal relationship found between the serum urate and an aortic dissection [123].

#### 4.1.3. Heart Failure

In epidemiological studies, an association between hyperuricemia and heart failure (HF) has been demonstrated [124]. A systematic review and meta-analysis revealed that hyperuricemia is linked to a 65% increased risk of developing HF. Moreover, for each 1 mg/dL (approximately 59 μmol/L) increase in the SUA levels, the odds of developing HF rise by 19% [125]. The evidence suggests that upregulated xanthine oxidase activity and the increased production of reactive oxygen species (ROS) are central to the pathogenesis of HF associated with hyperuricemia [124]. This aligns with the aforementioned finding that metabolic hyperuricemia appears to be more detrimental than renal hyperuricemia—the excessive production of uric acid in metabolic hyperuricemia would lead to the higher coproduction of ROS, whereas in renal hyperuricemia, there is no overproduction of uric acid, but the SUA is elevated due to underexcreting kidneys.

#### 4.1.4. Atrial Fibrillation

Studies have shown that elevated SUA levels and gout are associated with an increased risk of atrial fibrillation (AF) [126,127,128,129]. Hyperuricemia is independently associated with an increased risk of all-cause mortality and hospitalization for heart failure in patients with AF [130]. A recent study by Wu et al. suggested that incorporating uric acid into the CHA2DS2-VASc score—recommended by guidelines for assessing the embolism risk in AF patients [131,132]—significantly improves the score’s ability to identify patients at a high risk for new-onset AF after acute myocardial infarction [133]. A Mendelian randomization analysis also found that higher SUA levels were associated with an increased AF risk and that the SLC17A1 gene may offer protective effects against AF. However, other gene variants and the weighted genetic risk score did not provide evidence of a causal relationship between the SUA and AF [134].

#### 4.1.5. Cerebrovascular Conditions

Two meta-analyses of prospective studies demonstrated a significant relationship between hyperuricemia and acute ischemic stroke [135,136]. Another meta-analysis revealed a significant dose–response relationship between elevated SUA levels and stroke risk, indicating that, for every 1 mg/dL (approximately 59 μmol/L) increase in the SUA, the risk of stroke rises by approximately 10% [137]. It has been demonstrated that hyperuricemia is linked to hemorrhagic stroke as well [138,139]. A recent nationwide retrospective cohort study in Taiwan examined the stroke risk in patients with gout. It found that the hazard ratio (HR) for stroke was lower in the gout group (0.92) compared to the control group during the first three years of follow-up. However, after three years, the HR for the gout group increased to 1.08, surpassing that of the control group. Similarly, the HR for hemorrhagic stroke was lower in the gout group during the initial three years (0.88), but rose to 1.14 after that period [140]. Severe hypertension has been implicated as a possible mediator in the relationship between hyperuricemia and stroke [141].

### 4.2. Chronic Kidney Disease

Gout and hyperuricemia are present in 25% and 60% of patients with CKD, respectively [142]. Under normal physiological conditions, approximately one third of uric acid is eliminated from the body through the intestines and two-thirds through the kidneys [143]. Therefore, kidney dysfunction leads to a buildup of uric acid in the blood through the underexcretion of SUA. Around half of patients with CKD develop hyperuricemia by the time they begin dialysis [144]. However, it has also been hypothesized that elevated SUA levels might be the cause of the worsening of kidney function [144]. Most Mendelian randomization studies have not demonstrated that genetic polymorphisms leading to elevated serum uric acid levels directly translate to an increased risk for CKD [142], so the causative role of hyperuricemia on the development of CKD remains a debatable issue.

### 4.3. Diabetes and Insulin Resistance

Previous studies have demonstrated a high prevalence of diabetes among individuals with hyperuricemia and/or gout. A recent meta-analysis of 38 studies reported wide variations in diabetes prevalence, ranging from 3% to 67% among patients with hyperuricemia and from 6% to 35% among those with gout. The combined diabetes prevalence was 19.1% in individuals with hyperuricemia and 16.7% in those with gout [145]. The prevalence of hyperuricemia in individuals with diabetes is also notably high, ranging between 25.3% and 33.8% [146,147,148,149,150,151]. Several Mendelian randomization studies investigating the relationship between circulating uric acid and type 2 diabetes mellitus (T2DM) found no evidence to support a causal effect of circulating uric acid on the risk of developing diabetes [152,153,154]. A Mendelian randomization analysis on the causal relationship between uric acid and diabetic macrovascular disease, however, provided evidence for a causal relationship between uric acid and diabetic macrovascular disease in females with T2DM, but not in males [155]. Even though the causal link remains unclear, it is well known that hyperuricemia is strongly associated with the development of diabetes and its long-term complications [156].

People with T2DM and prediabetes display varying levels of insulin resistance [157], a condition characterized by elevated blood sugar levels and the body’s compensatory response of producing more insulin [158]. In the prediabetic stage, insulin resistance is the strongest predictor of the future development of T2DM [159]. Hyperuricemia has been identified as an independent risk factor for insulin resistance in healthy young individuals [160]. A positive association between SUA levels and insulin resistance has also been shown in older non-diabetic individuals [161]. It has been shown that elevated SUA levels often precede the onset of insulin resistance [162], suggesting a potential link between the two. However, the cause–effect relationship remains unclear. While some studies suggest that insulin resistance may contribute to the development of hyperuricemia [163,164,165], the exact mechanisms are still under investigation. A Mendelian randomization analysis investigating the relationship between plasma uric acid and insulin resistance in newly diagnosed T2DM patients found an association between elevated SUA levels and an increased risk of insulin resistance. This association was more pronounced in women than in men. However, the study did not support a causal role of plasma uric acid in insulin resistance among these patients [166].

### 4.4. Steatotic Liver Disease

Several studies have reported an association between elevated SUA levels and steatotic liver disease [167,168,169]. It has been shown that individuals with metabolic hyperuricemia have a higher fatty liver index—a reliable surrogate marker for fatty liver disease [170] —compared to those with renal hyperuricemia [171]. While there is clear evidence of an increased SUA across various metabolic dysfunction-associated steatotic liver disease (MASLD) cohorts, the evidence for a causal relationship remains weak [172]. A Mendelian randomization study found no evidence for a causal link between the SUA and MASLD [173]. Conversely, another Mendelian randomization analysis suggested that MASLD could causally increase the SUA levels, but it did not confirm a causal association of SUA levels with the risk of MASLD [174].

### 4.5. Osteoarthritis

Epidemiologic links between gout and osteoarthritis (OA) have been observed, though the nature of this relationship—whether gout predisposes to OA or vice versa—has been infrequently studied. In a UK case-control study of 39,111 patients with incident gout and matched controls, the risk of developing OA was 45% higher in subjects with gout. Additionally, those with gout were 27% more likely to have had a prior diagnosis of OA compared to controls [175]. It was found in another study that asymptomatic hyperuricemia (elevated uric acid without a history of gout attacks) was associated with a greater knee OA severity, and the presence of gout was correlated with even more severe knee OA, suggesting a dose–response relationship [176]. However, another study found no significant association between gout and knee OA, though it did report a link between gout and foot OA [177]. Additionally, one study noted that the presence of osteophytes—hallmarks of OA—was associated with hyperuricemia in women, but not men, even after adjusting for factors like BMI [178]. A cross-sectional study of 92 patients with tophaceous gout in the feet reported that joints with MSU crystal deposition, detected via DECT, were significantly more likely to exhibit the features typical of osteoarthritis—such as osteophytes (odds ratio: 3.9), subchondral sclerosis (odds ratio: 6.9), and joint space narrowing (odds ratio: 4.2)—compared to joints without MSU deposits. These features are characteristic of OA, but not typically associated with gout [179].

Associations between so-called asymptomatic hyperuricemia and musculoskeletal pain have been observed [180,181], and recent findings demonstrate that individuals with asymptomatic hyperuricemia purchase more prescription analgesics than normouricemic individuals [182]. This suggests that hyperuricemic individuals without a history of gout attacks may not be truly asymptomatic, calling into question the validity of the term “asymptomatic hyperuricemia”. It is highly likely that the pain experienced by individuals with non-gouty hyperuricemia is largely attributable to osteoarthritis, although strong evidence to support this is still lacking.

It has been observed that gout increases the risk of incident total joint replacement [183]. However, the effect of hyperuricemia on the risk of incident joint replacement has not yet been investigated.

### 4.6. Respiratory Disease

Obstructive sleep apnea (OSA) is linked to hyperuricemia due to hypoxia-induced nucleotide turnover [184]. This might explain why gout flares are 2.4 times more common during the night and early morning than during the day [185]. OSA has been shown to independently increase the risk of developing gout [186,187,188]. Additionally, so-called asymptomatic hyperuricemia is associated with OSA [189,190]. The findings from a bidirectional two-sample Mendelian randomization study suggest that OSA is causally associated with elevated SUA levels, but not independently with gout risk [191]. Early management of comorbid obstructive sleep apnea may help lower the risk of premature mortality in individuals with gout and hyperuricemia [192].

A study by Yang et al. explored the relationship between SUA levels and lung function in individuals with and without chronic obstructive pulmonary disease (COPD). The findings revealed that elevated SUA levels were associated with lower lung function, particularly in COPD patients [193]. Additionally, it has been shown that hypoxia resulting from impaired lung function increases uric acid production, and hyperuricemia is correlated with higher rates of COPD exacerbations and COPD-related mortality [194]. It has been suggested that uric acid-induced inflammation may contribute to the activation and proliferation of inflammatory cells in the respiratory epithelium, potentially involving endothelin-1. Endothelin-1 has been shown to upregulate inflammatory mediators such as IL-6 and IL-8 and has been linked to increased mucus production, airway edema, and bronchial hyperresponsiveness [195].

### 4.7. Eye Disease

Gout has been linked to an increased risk of dry eye disease [196] and age-related macular degeneration [197,198], though further research is needed to establish causal connections.

A recent meta-analysis indicated that glaucoma patients tend to have higher SUA levels compared to controls, but the difference was not statistically significant [199]. Conversely, a study by Bhat et al. found decreased SUA levels in patients with primary open-angle glaucoma (POAG) compared to healthy controls, and the study also identified a significant negative association between the SUA levels and the SUA-to-creatinine ratio with the severity of POAG [200].

A meta-analysis investigating the association between gout and cataract risk suggested that gout may be linked to a higher likelihood of age-related cataracts [201]. In a study by Qin et al., elevated uric acid levels in the aqueous humor were found to be associated with posterior subcapsular cataracts in human lenses [202]. It has been reported that the uric acid-driven activation of the NLRP3 inflammasome can trigger lens epithelial cell senescence, contributing to cataract formation [203].

## 5. Clinical Implications

A summary of the clinical implications related to the risk factors and comorbidities of gout and hyperuricemia is presented in Table 2.

Even though gout is often perceived as an inflammatory joint disease, it is crucial to recognize it as a systemic condition affecting more than just the joints. Numerous comorbidities are associated with gout, making it essential to adopt a holistic approach to managing the condition. Every gout patient should undergo a cardiovascular assessment. Given the established link between gout and cardiovascular diseases, as well as cardiovascular mortality, co-existing cardiovascular conditions should be managed concurrently. Managing other cardiovascular risks, such as hypertension, hyperlipidemia, and diabetes, is vital in gout patients. Screening for associated comorbidities and cardiovascular risk factors has been highlighted as one of the overarching principles in The European Alliance of Associations for Rheumatology (EULAR) recommendations for gout management [204].

In clinical practice, addressing the modifiable risk factors of hyperuricemia and gout can benefit patients beyond reducing gout flares, improving their overall health, well-being, and longevity. Weight loss in overweight individuals has been shown to reduce both the risk and frequency of gout attacks [205]. Both EULAR and The American College of Rheumatology (ACR) recommend weight loss in individuals with gout to manage the condition and reduce the risk of flares [204].

Dietary modifications should also be considered. Patients with gout and hyperuricemia should be advised to limit purine-rich foods, such as red meats, seafood, and legumes, and to avoid sugar-sweetened drinks and foods rich in fructose. Alcohol avoidance should be encouraged as well.

Although the relationship between physical activity and gout is not fully understood, regular exercise benefits individuals with gout and hyperuricemia and promotes overall health. The EULAR guidelines for gout management recommend regular physical activity for gout patients [204].

Medications prescribed for other conditions can impact SUA levels, and this should be considered when prescribing treatment. The ACR guidelines suggest switching hydrochlorothiazide to alternative antihypertensive medications when feasible for patients with gout. Losartan is conditionally recommended as a preferred antihypertensive agent in gout patients [206]. SGLT2 inhibitors have been shown to significantly lower SUA levels and reduce the risk of gout incidence and flares [207,208]. Although not included in current gout management guidelines, SGLT2 inhibitors should be considered in hyperuricemic patients with indications such as T2DM, CKD, or HF.

The EULAR recommendations advocate for pharmacological treatment with ULT in patients with recurrent gout flares (≥2/year), tophi, urate arthropathy, and/or renal stones [204]. The ACR guidelines recommend ULT for patients who have experienced more than one gout flare, or have tophi, radiographic joint damage, CKD stage ≥3, or SUA levels ≥9 mg/dL (535 µmol/L) [206].

The current guidelines generally do not recommend treating asymptomatic hyperuricemia due to insufficient data supporting a favorable benefit-to-risk ratio [206,209,210]. However, the Polish Society of Hypertension guidelines recommend lowering the SUA level to 5.0 mg/dL (300 μmol/L) or below in hypertensive patients with high cardiovascular risk [211]. The Japanese guidelines have advocated for the treatment of asymptomatic hyperuricemia for over a decade, recommending treatment for SUA levels ≥9.0 mg/dL (535 µmol/L) without complications, or ≥8.0 mg/dL (476 µmol/L) with complications such as kidney disease, CVD, diabetes, or metabolic syndrome [17].

Recent findings suggest that the underlying cause of hyperuricemia influences the mortality and morbidity risks [97,98,171]. This makes it plausible that the benefit of ULT could be greater in patients with metabolic hyperuricemia compared to those with renal hyperuricemia. However, clinical trials investigating the potential benefits of ULT in hyperuricemic individuals without a history of gout are lacking, and further research is needed to determine whether ULT should be prescribed in cases of metabolic hyperuricemia without gout attacks. Nonetheless, individuals with metabolic hyperuricemia should receive appropriate treatment for underlying metabolic conditions that may contribute to hyperuricemia (e.g., obesity, OSA, diabetes), along with advice on dietary modifications.

## 6. Conclusions

Gout and hyperuricemia are increasingly prevalent metabolic conditions, contributing to a growing healthcare burden worldwide. It is crucial for clinicians to recognize the systemic nature of these conditions, which are associated with numerous comorbidities and an elevated risk of mortality. A holistic approach to management is essential, one that not only addresses acute gout flares, but also emphasizes lifestyle modifications and the comprehensive treatment of comorbid conditions affecting multiple organs and systems. Such an approach will provide the greatest benefit to patients and help mitigate the broader health implications of gout and hyperuricemia.

## Figures and Tables

**Table 1:** Risk factors for gout and/or hyperuricemia.

Non-Modifiable Risk Factors	Comments	Modifiable Risk Factors	Comments
**Age**	The prevalence of hyperuricemia and gout rises with age	**Body composition**	Obesity and abdominal adiposity increase the risk of hyperuricemia and gout; weight loss has a protective effect against gout
**Sex**	Hyperuricemia and gout are more prevalent in men; in postmenopausal women, however, the SUA levels are close to those of men of the same age	**Dietary factors**	Dietary factors that increase the risk of hyperuricemia and/or gout: Purine-rich foods (seafood, legumes, red meat) Alcohol Sugar-sweetened beverages and a high-fructose diet Dietary factors that decrease the risk of hyperuricemia and/or gout: A plant-based diet Coffee Tea Dried fruit Cherries Milk and dairy products
**Genetic factors and ethnicity**	Over 20 susceptibility genes for hyperuricemia and gout have been identified Gout appears to be more prevalent in Black and Asian individuals than in White individuals	**Medication**	Medications that increase SUA levels Diuretics (especially thiazide diuretics) Beta blockers Low-dose ASA Pyrazinamide Ethambutol Calcineurin inhibitors Insulin Testosterone Medications that decrease SUA levels Losartan SGLT2 inhibitors ACE inhibitors High-dose ASA Calcium channel blockers Statins Fenofibrate Leflunomide Estrogen therapy

SUA, serum uric acid; ASA, acetylsalicylic acid; SGLT2, sodium glucose co-transport 2; ACE, angiotensin-converting enzyme.

**Table 2:** Clinical implications related to the risk factors and comorbidities of gout and hyperuricemia.

Factors That Need to Be Addressed	Comments
**Cardiovascular risks**	Every patient should undergo a cardiovascular assessment Modifiable cardiovascular risks (smoking, hypertension, high cholesterol levels, diabetes, pre-diabetes) should be managed according to relevant guidelines Any cardiovascular diseases co-existing with hyperuricemia and gout should be treated appropriately
**Other comorbidities**	In patients with hyperuricemia or gout, questions about symptoms of OSA should be asked, and additional testing should be conducted if symptoms are present The GFR should be measured, and any underlying renal conditions should be treated; patients with CKD stage 3–4 may benefit from SGLT2 inhibitors, as these drugs not only slow the progression of renal impairment, but also help reduce SUA levels and the frequency of gout flares Patients with MASLD should be advised on lifestyle changes to slow disease progression, including weight loss, increased physical activity, and reducing their intake of saturated fats and high-sugar foods. Those with advanced disease (e.g., hepatic fibrosis or cirrhosis) should be referred to a gastroenterologist
**Weight**	Weight loss in overweight individuals has been shown to lower SUA levels and reduce both the risk and frequency of gout attacks
**Physical activity**	Patients should be encouraged to engage in regular physical activity; it has been shown to decrease the excess mortality associated with chronic hyperuricemia
**Dietary factors**	All patients with gout or hyperuricemia should receive dietary guidance, including limiting purine-rich foods (such as red meat, seafood, and legumes), avoiding sugar-sweetened drinks and high-fructose foods, and reducing alcohol intake
**Medications prescribed for indications other than treating hyperuricemia**	The medications prescribed for patients with gout or hyperuricemia should be regularly reviewed When feasible, thiazide diuretics should be switched to alternative antihypertensive medications; losartan is the preferred choice for patients with gout or hyperuricemia In hyperuricemic patients with conditions such as T2DM, CKD, or HF, SGLT2 inhibitors should be considered

OSA, obstructive sleep apnea; GFR, glomerular filtration rate; CKD, chronic kidney disease; SGLT2, sodium glucose co-transport 2; SUA, serum uric acid; MAFLD, metabolic dysfunction-associated steatotic liver disease; T2DM, type 2 diabetes mellitus; HF, heart failure.

## Data Availability

No new data were created or analyzed in this study. Data sharing is not applicable to this article.
